# Predictors of fibromyalgia: a population-based twin cohort study

**DOI:** 10.1186/s12891-016-0873-6

**Published:** 2016-01-15

**Authors:** Ritva A. Markkula, Eija A. Kalso, Jaakko A. Kaprio

**Affiliations:** Anaesthesiology, Intensive Care and Pain Medicine, University of Helsinki and Helsinki University Hospital, Helsinki, Finland; Faculty of Medicine, University of Helsinki, Helsinki, Finland; Department of Public Health, Hjelt Institute, University of Helsinki, Helsinki, Finland; Institute for Molecular Medicine, University of Helsinki, Helsinki, Finland; Department of Mental Health and Alcohol Abuse Services, National Institute for Health and Welfare, Helsinki, Finland

## Abstract

**Background:**

Fibromyalgia (FM) is a pain syndrome, the mechanisms and predictors of which are still unclear. We have earlier validated a set of FM-symptom questions for detecting possible FM in an epidemiological survey and thereby identified a cluster with “possible FM”. This study explores prospectively predictors for membership of that FM-symptom cluster.

**Methods:**

A population-based sample of 8343 subjects of the older Finnish Twin Cohort replied to health questionnaires in 1975, 1981, and 1990. Their answers to the set of FM-symptom questions in 1990 classified them in three latent classes (LC): LC1 with no or few symptoms, LC2 with some symptoms, and LC3 with many FM symptoms. We analysed putative predictors for these symptom classes using baseline (1975 and 1981) data on regional pain, headache, migraine, sleeping, body mass index (BMI), physical activity, smoking, and zygosity, adjusted for age, gender, and education. Those with a high likelihood of having fibromyalgia at baseline were excluded from the analysis. In the final multivariate regression model, regional pain, sleeping problems, and overweight were all predictors for membership in the class with many FM symptoms.

**Results:**

The strongest non-genetic predictor was frequent headache (OR 8.6, CI 95 % 3.8–19.2), followed by persistent back pain (OR 4.7, CI 95 % 3.3–6.7) and persistent neck pain (OR 3.3, CI 95 % 1.8–6.0).

**Conclusions:**

Regional pain, frequent headache, and persistent back or neck pain, sleeping problems, and overweight are predictors for having a cluster of symptoms consistent with fibromyalgia.

**Electronic supplementary material:**

The online version of this article (doi:10.1186/s12891-016-0873-6) contains supplementary material, which is available to authorized users.

## Background

Fibromyalgia (FM) is today better understood and recognised than 25 years ago, when the classification criteria for FM were first published [1]. These criteria are: (1) A history of widespread pain (WSP) (pain on the left and right sides of the body, above and below the waist, and axial skeletal pain); (2) pain on palpation of at least 11 of 18 specified sites. Until then, the syndrome was ill-defined, with its very existence questioned by many. Even now, we do not still fully understand the reasons for the development of FM and its symptoms, which makes prevention impossible.

Prospective population-based studies on the incidence and predictors of FM are few, whereas many cross-sectional and tertiary clinic studies assess predictors. In a cross-sectional setting, the sequential relationship remains unclear, and one can only identify an association, not a prediction. Among tertiary care patients, selection bias may significantly alter the profile and aggregation of predictors. So far, the few prospective population-based studies on the incidence and predictors of FM or widespread pain have focused on one or a few potential predictors. Regional back pain as well as depressive tendencies predicted WSP in schoolchildren [2] and FM in adults [3]. Another rather small study revealed higher age and multiple pain sites at baseline to be risk factors for WSP [4]. One prospective population-based study indicated an independent association between a high BMI and risk for FM. In the same study, leisure-time physical exercise level and future FM tended towards a weak inverse dose–response association [5]. Sleep disturbance is associated with increased sensitivity to noxious stimuli and with chronic pain intensity level [6–9]. Two large prospective studies revealed an association between poor sleep and FM [10] or WSP [11], the latter also showing association between low BMI, anxiety, depression and WSP.

Genetic differences accounted for approximately 50 % of the variability of FM-associated symptoms in our large twin-population-based cross-sectional sample, using a latent class approach for symptom classification [12]. Moreover, many disease-related predictors have a genetic component reflecting individual genetic differences in the propensity to acquire the predictor (such as smoking, alcohol use) or the propensity in the degree to which it is expressed (such as education or physical activity). This has been shown by family and twin studies, and increasingly confirmed by genome-wide association studies. Thus, genetic factors shared by FM and putative predictors may also generate an association without being causal. Studies of trait-discordant twin pairs (i.e. one co-twin has a trait, and the other does not) permit controlling of such genetic confounds by examining within-pair associations of the predictor and future disease. Twins share their genetic background, either fully (monozygotic twins) or in 50 % of their segregating genes (dizygotic twins). In addition, twins usually also share family and childhood exposures.

The aim of this study was to evaluate simultaneously, in a population-based large prospective twin cohort, several potential predictors for fibromyalgia and possible genetic confounds, these factors having been assessed 9–15 years prior to the evaluation of FM symptoms.

## Methods

### Study population

The study population is based on the Finnish Twin Cohort, which consists of twin pairs born before 1958, with both twins alive in 1975, at the time of the first cohort-wide health questionnaire [13]. A second cohort-wide health questionnaire was sent in 1981, and a follow-up questionnaire was mailed in 1990 to the 16,179 twins born in 1930–1957 who had replied to either of the previous questionnaires. Of these twins, 12,502 subjects (77.3 %) responded [14]. For the first two questionnaires, response rates were 89 and 84 %, respectively.

The study is limited to questionnaire and anonymised medical record data, for which permission from the relevant authorities has been sufficient. The use of the questionnaire data for record linkage studies has been approved by the ethics committee of the Department of Public Health, University of Helsinki. The twin cohort members were informed about the use of the questionnaire information to study genetic and environmental influences on common diseases and their predictors and also about the record linkage, and been informed that they may withdraw from the study at any time.

### The screening method for an epidemiological estimation of subjects with potential fibromyalgia

In our earlier study [12], based on the 1990 survey data, we classified these subjects into three latent symptom classes based on their answers to certain questions aiming to detect fibromyalgia. The latent class (LC) analysis is a statistical system which classifies data in categories that are internally as homogeneous and externally as heterogeneous as possible in relation to specific qualities, in this case to these FM-questions. From the original survey questions based on the Yunus et al. criteria suggestions from 1989 [15], we chose these questions about FM symptoms and FM-associated symptoms (from now on all called FM symptoms) according to the American College of Rheumatology 1990 classification criteria [1]. The questions were: How often have you had these symptoms during the last year? 1) stiffness in the limbs and trunk in the morning, 2) stiffness in the limbs and trunk in the evening, 3) pain and stiffness in the neck, 4) soreness with touching of the neck, back, trunk, or limbs, 5) numbness in the extremities, 6) daytime tiredness. 7) Does the stiffness in the limbs and trunk in the morning last “less than 15 min”, “about half an hour”, or “about 1–2 h”? and 8) Does low air pressure (rain, snow, storm) worsen the pain in the trunk or limbs?

Questions 1 to 6 were provided with the alternatives “never”, “daily or nearly daily”, “3–5 times per week”, “1–2 times per week”, “about once a month”, and “more seldom”. Exclusion of subjects with missing data concerning these classification questions yielded 10,608 subjects. Three latent classes represented the subjects best: LC1 indicated no or few symptoms; LC2 had some symptoms; and LC3 had a high frequency of FM symptoms resembling clinical FM patients. In LC3, over 80 % of the subjects had morning stiffness, 58 % had tender points, 65 % had neck pain and stiffness, and 48 % had daytime tiredness at least three times a week.

We also used the answers of a group of 49 clinically-diagnosed FM patients [12] as a validation data set. The LC method described classified all clinical FM patients to LC3 [12].

### Exclusions

**Fig. 1:**
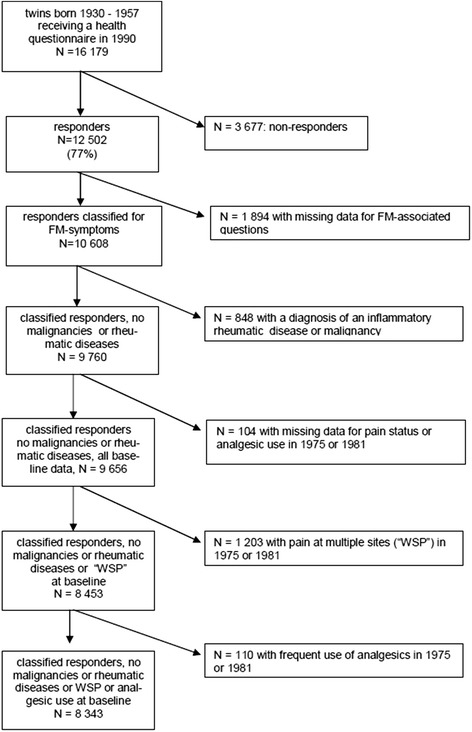
Flow chart of the study. *WSP* widespread pain

In the present study, we aimed to identify predictors of onset of LC2 and, in particular, LC3 memberships, i.e. predictors of the high incidence of FM symptoms as a proxy for WSP (widely used as a screening method) and FM. As these symptoms were not assessed in the 1975 and 1981 questionnaires, we used available information on pain-associated conditions to exclude those who would very likely be classified into LC2 or 3 as shown in the flowchart (Fig. 1). Thus, we first excluded the subjects having inflammatory rheumatic diseases or malignancies (which might cause the symptom load) by linking the data with national Special Refund Category data in the Drug Reimbursement Register held by the Social Insurance Institution and with data on incident cancers from the Finnish Cancer Registry to 1993. Subjects with missing data concerning particular regional pain questions either in 1975 or 1981 were excluded. We then excluded the subjects with possible FM symptoms at baseline by excluding those who reported pain in the neck and shoulders and back at the same time, in either 1975 or 1981 (this served as a proxy for pain at multiple sites, referred to as “WSP” in Fig. 1). Earlier, we had shown that regular use of analgesics was almost entirely restricted to the LC3 subjects. Therefore we also excluded those who reported having used analgesics of that frequency (on at least 180 days per year) in either 1975 or 1981 (Fig. 1). The final baseline sample for analysis, i.e. the population at risk, we presumed to be free of pain at multiple sites and free of major conditions giving rise to similar symptoms.

### Study variables

We selected the variables analysed as potential predictors based on reports in the literature on putative predictors; that is, variables that had positive associations with FM or WSP in either cross-sectional or prospective, population-based, qualified studies mentioned in this article [2–11]. These included separate regional pain symptoms, headache, migraine, sleeping, underweight, overweight or obesity as indexed by BMI, smoking, and physical activity.

These potential predictors were assessed as follows. A simple question “In the last years, have you had pain in the back, shoulders, or neck that has impaired your working capacity?” with the alternatives “yes” and “no” for each region, assessed regional pain symptoms. The occurrence of migraine was based on the subjects’ report of diagnosed migraine [16]. The occurrence and frequency of headache was assessed in 1981 only by the question “Do you have headache?” with the options “daily or almost daily”, “many times a week”, “about once in a week”, “about once a month”, “many times in a year (but not every month)”, “once in a year or less” and “practically never” (reference category) [16]. Another question was “Do you usually sleep well?” with the options “well”, “fairly well”, “fairly badly”, “badly”, and “cannot answer” [17]. As the number of individuals reporting sleeping “badly” at baseline was small (36 and 52 in 1975 and 1981), we merged the two first alternatives into the category “good sleep” (reference category) and the next two into “poor sleep”. The number of “cannot answer” replies was also small (44 and 48 in 1975 and 1981) and they were handled as missing data.

The self-reported height and weight in both 1975 and 1981 produced BMI values which were classified in four categories based on WHO criteria: at least 30 (obese), at least 25 but less than 30 (overweight), under 18.5 (underweight), and at least 18.5 but under 25 (normal weight, reference category).

Two questions were on leisure-time physical activity. The question about year-round leisure-time activity, with the alternatives 1) “I do not exercise in my leisure time practically at all”, 2) “a bit”, 3) “fairly”, 4) “fairly much” and 5) “much”, originally assessed physical activity. As we wanted to look at physical passivity as a possible predictor and activity as a potential protecting factor, we re-classified these replies into three categories: physically passive (1–2), physically active (4–5), and average (3), which was used as a reference. Leisure-time exercise frequency was originally assessed by the alternatives 1) less than once, 2) 1–2 times, 3) 3–5 times, 4) 6–10 times, 5) 11–19 times, and 6) more than 20 times a month [18]. For this study we re-categorised these as three alternatives: 1) at most twice, 2) 3–10 times (reference category), and 3) at least 11 times a month. For smoking status, the subjects were classified into four groups: current smoker, former smoker, occasional smoker, and non-smoker (reference category) [19].

Reported gender, age (calculated from registry data and the date of response to the query), and education presented potential confounders. Education was originally reported with nine alternatives from elementary school to college or university degree. For this study, we calculated the mean of the education years for each alternative to form a continuous variable.

Zygosity was diagnosed by a validated questionnaire method using questions on similarity in appearance and confusion by strangers [20].

### Statistical analysis

Two sets of analyses were conducted, first among all individuals (as a standard cohort analysis) and secondly within twin pairs to adjust for unmeasured genes and other factors shared by siblings.

#### Cohort analysis of individuals

For the analysis of potential predictors for FM symptoms among individuals, we used multinomial logistic regression analysis with the three latent symptom classes as the categories of the dependent variable. The asymptomatic class, LC 1, served as the reference category. As potential predictors, we analysed back pain, neck pain, shoulder pain, headache (data available only from 1981), migraine (data available only from 1981), sleeping problems, physical passivity or activity, BMI, and smoking, using the information from 1975 to 1981. The bivariate associations were first tested with logistic regression analysis, adjusting for age and gender. Based on these analyses, (i.e. including those variables with significant associations), we performed multivariate logistic regression analyses, adjusted for gender, age, and education. These variables were taken forward to the within family, i.e. pairwise analyses described below.

Before the pairwise analyses, we used the standard cohort approach to test for any possible moderation effect. We thus included interaction terms for gender, age, and education in the analysis with the data stratified by gender (men vs. women), age (under vs. over the median age), and education (high-school education, yes vs. no).

#### Post-hoc analysis of individuals

To analyse whether the persistence or recurrence of regional pain (back, shoulder, and neck pain) had any effect on the association with the future symptom class, we made re-analyses in a sub-sample with those individuals who had replied to the questionnaires both in 1975 and 1981, comparing positive reports at both time points (persistent or recurrent pain) to positive–negative combinations (pain at only one time point) and negative reports at both time points (no pain) in all three regions.

We did an additional analysis considering only those with more recent pain onset by inclusion of those subjects reporting no pain (back, shoulder, or neck) in 1975.

#### Within-pair analysis

The second set of analyses used the information on twinship to assess the possible effect of genetic or familial environmental factors on the relationship between predictors and FM symptoms. We identified the twin pairs discordant for “latent class” status, i.e. pairs in which one twin was classified as LC3 and the co-twin as LC1. If the genetic or environmental factors shared by the twin pair could account for the relationship between predictors and FM symptoms, the risk (the ORs) would presumably decrease. If particularly the genetic factors could account for the relationship, some association would appear in dizygotic (DZ) twins (who share on average 50 % of their segregating genes) but not in monozygotic (MZ) twins (who have an identical genotype) [21]. For this assessment, we used conditional logistic regression analysis. The predictors that were significant in the multivariate model were all included in this analysis.

#### Statistical software

We used the Stata version 12 in the pairwise analyses; in all other analyses we used SPSS version 19.

## Results

The original sample without missing data after excluding those fulfilling the disease exclusion criteria (inflammatory rheumatic diseases and malignancies) consisted of 9656 individuals, of which 46.2 % were men and 53.8 % women. Of these individuals, 11.7 % were classified in 1990 into latent class 3 (with a high frequency of FM symptoms). In 1975, 4.6 % individuals reported pain in both back, shoulders, and neck, and in 1981 this proportion was 10.1 %. After exclusion of these subjects with possible FM symptoms and those with frequent use of analgesics at the baseline, we had a final sample of 8343 subjects (Fig. 1).

The final sample comprised 3946 (47.3 %) men and 4397 (52.7 %) women, with an overall mean age in 1975 of 27.7 ± 7.3 years. Some gender differences for the covariates emerged as expected. All subsequent analyses were adjusted for gender.

In 1990, 700 individuals (8.4 %) in this sample were classified into latent class 3 with a high frequency of FM symptoms, 2501 individuals (30.0 %) into latent class 2 with some symptoms, and 5142 individuals (61.6 %) into latent class 1 with no or few symptoms. Baseline characteristics in terms of the potential predictor variables of this study varied across these latent classes from LC1 to LC3: the frequency of all of the proposed predictors increased, whereas the mean value of the confounding factor education decreased (Additional file 1).

### Cohort analysis of individuals

In the univariate regression analyses adjusted for age and gender, all of the proposed predictors significantly predicted membership in the high-frequency symptom class. Therefore, all the variables were included in the multivariate analysis. In the final multivariate model, however, only regional pain problems, sleeping problems, and overweight remained significant predictors (Table 1 and Additional file 2). A nearly constant “dose response” is evident with regard to the statistically significant predictors, because the predictors were more strongly associated with LC3 than with LC2.

**Table 1:** Predictors of fibromyalgia symptoms: multivariate analyses

	OR (95 % CI) for LC2 1975 variable data	OR (95 % CI) for LC3 1975 variable data	*n* by variable in 1975	OR (95 % CI) LC2 1981 variable data	OR (95 % CI) LC3 1981 variable data	*n* by variable in 1981
age (years)	1.02 (1.01–1.03)	1.06 (1.05–1.07)	8343	1.02 (1.01–1.03)	1.06 (1.05–1.08)	8343
gender (female/male)	1.34 (1.20–1.50)	1.41 (1.17–1.70)	8343	1.08 (0.94–1.23)	1.16 (0.89–1.49)	8343
back pain (yes/no)	1.56 (1.38–1.76)	2.26 (1.88–2.72)	8149	1.73 (1.47–2.04)	2.99 (2.31–3.88)	7281
shoulder pain (yes/no)	1.42 (1.14–1.77)	1.84 (1.36–2.49)	8146	1.77 (1.41–2.21)	1.75 (1.23–2.48)	6636
neck pain (yes/no)	1.94 (1.60–2.35)	2.34 (1.79–3.07)	8144	1.23 (0.98–1.54)	1.76 (1.26–2.46)	6718
poor sleep	1.27 (0.96–1.66)	1.78 (1.22–2.60)		1.58 (1.17–2.14)	2.34 (1.49–3.67)	
good sleep	1.00 (reference category)	1.00	8116	1.00	1.00	7966
BMI (kg/m-squared)						
≥ 30	1.19 (0.78–1.80)	1.86 (1.09–3.17)		1.19 (0.81–1.76)	1.65 (0.91–3.01)	
25–29.9	1.20 (1.03–1.40)	1.60 (1.28–1.99)		1.32 (1.12–1.56)	1.67 (1.27–2.20)	
18.5–24.9	1.00 (reference category)	1.00		1.00	1.00	
< 18.5	0.80 (0.64–1.00)	0.86 (0.55–1.33)	8056	0.62 (0.44–0.88)	0.56 (0.25–1.25)	7913
education (years)	0.96 (0.94–0.97)	0.86 (0.83–0.90)	8134	0.97 (0.95–0.98)	0.89 (0.85–0.92)	7754
physical activity						
passive	0.94 (0.83–1.07)	1.15 (0.93–1.43)		1.04 (0.90–1.21)	1.08 (0.82–1.41)	
moderate	1.00 (reference category)	1.00		1.00	1.00	
active	0.90 (0.76–1.08)	1.00 (0.73–1.38)	8148	0.88 (0.72–1.08)	0.87 (0.58–1.30)	8007
exercise frequency/month						
1–2 times	1.02 (0.90–1.16)	0.98 (0.79–1.20)		0.96 (0.82–1.13)	0.94 (0.70–1.26)	
3–10 times	1.00 (reference category)	1.00		1.00	1.00	
> 11 times	0.856 (0.74–1.00)	0.91 (0.69–1.18)	7889	1.01 (0.85–1.20)	1.01 (0.73–1.40)	7849
smoking						
current	1.10 (0.97–1.24)	1.20 (0.98–1.47)		1.11 (0.95–1.29)	1.21 (0.91–1.59)	
former	1.14 (0.98–1.32)	0.97 (0.75–1.25)		1.05 (0.90–1.24)	0.95 (0.70–1.30)	
occasional	1.27 (0.97–1.66)	0.90 (0.53–1.52)		1.12 (0.79–1.58)	0.83 (0.40–1.72)	
never	1.00 (reference category)	1.00	8146	1.00	1.00	7936
migraine (yes/no)	n.a.	n.a.		0.86 (0.68–1.08)	1.17 (0.82–1.67)	7986
headache frequency	n.a.	n.a.				
many /week				3.35 (2.19–5.13)	7.22 (4.03–12.95)	
1–4 /month				1.95 (1.62–2.35)	2.22 (1.56–3.15)	
some/year				1.64 (1.40–1.93)	1.64 (1.19–2.26)	
never				1.00 (ref. category)	1.00	7767

The reference category for the outcome is LC1, i.e. those with no or very few fibromyalgia symptoms [12]. Odds ratios and 95 % confidence intervals are provided for LC2 (with some fibromyalgia symptoms) and LC3 (with many fibromyalgia symptoms), based on latent class (LC) analyses in 1990. Predictors are assessed from data collected in 1975 and 1981 with the exception of migraine and headache frequency from 1981 only

While two of the age group-regional-pain interaction comparisons were nominally significant at the *p* < 0.05 level, neither survived adjustment for multiple testing, given that we had no a priori hypothesis that such interactions existed specifically for any predictor. Thus, we have no evidence that predictive value varies by level of these three covariates.

Most of the subjects in this sample, 5894 individuals, had replied to the questions concerning regional pain in the back, neck, or shoulders both in 1975 and in 1981, with most of the missing data in 1981 (Table 1). Of these individuals, 86.9 % reported no neck pain, 87.9 % reported no shoulder pain, and 74.1 % no back pain at either time point, whereas 5.9 % (349 individuals) reported back pain, 2.1 % (123 individuals) neck pain, and only 1.5 % (89 individuals) shoulder pain at both time points. These individuals may represent those with persistent regional pain. In the univariate analyses of this sub-sample, persistent regional pain in either back, neck, or shoulder was about twice as powerful a predictor of FM symptoms as was transient pain (Table 2). Only frequent headache was a stronger predictor than was persistent regional pain. Current smoking in 1981 was a significant predictor as well. In the multivariate analyses of 1981, poor sleep (OR = 2.23, 95 % CI 1.39–3.58) and high BMI (obesity OR = 1.72, 95 % CI 0.94–3.2 and overweight OR = 1.55, 95 % CI 1.17–2.05) remained predictors (data not shown). Headache remained a strong predictor with no change in the odds ratios, when the persistent regional pain variables were taken into the model.

**Table 2:** Predictors for fibromyalgia symptoms in the sub-sample with regional pain data from both 1975 and 1981 (5894 individuals)

	Univariate analyses^a^	Multivariate analyses	
	OR (95 % CI) for LC3	OR (95 % CI) for LC3 1975 variable data	OR (95 % CI) for LC3 1981 variable data
	*n* = 374 “cases”/ 5894 persons	*n* = 353 “cases”	*n* = 346 “cases”
headache frequency 1981		not assessed	
many /week	11.33 (6.63–19.35)		6.79 (3.73–12.37)
1–4 /month	2.66 (1.91–3.69)		2.06 (1.44–2.94)
some/year	1.78 (1.31–2.41)		1.53 (1.11–2.13)
never	1.00 (reference category)		1.00
back pain (yes/no)			
both 1975 and 1981	6.33 (4.55–8.81)	5.20 (3.67–7.38)	4.67 (3.28–6.67)
either 1975 or 1981	2.28 (1.77–2.94)	1.97 (1.51–2.59)	1.85 (1.41–2.44)
never	1.00 (reference category)	1.00	1.00
shoulder pain (yes/no)			
both 1975 and 1981	4.65 (2.41–8.97)	2.10 (1.02–4.34)	1.89 (0.89–4.02)
either 1975 or 1981	2.38 (1.77–3.19)	1.56 (1.12–2.16)	1.51 (1.08–2.12)
never	1.00 (reference category)	1.00	1.00
neck pain (yes/no)			
both 1975 and 1981	5.78 (3.42–9.75)	3.80 (2.11–6.83)	3.32 (1.83–6.02)
either 1975 or 1981	2.23 (1.66–2.99)	1.69 (1.22–2.33)	1.51 (1.09–2.09)
never	1.00 (reference category)	1.00	1.00

The reference category for the outcome is LC1, i.e. those with no or very few fibromyalgia symptoms [12]. Odds ratios and 95 % confidence intervals are provided for LC3 (with many fibromyalgia symptoms), based on latent class (LC) analyses in 1990. Predictors are assessed from data collected in 1975 and 1981 with the exception of headache frequency, which was only from the 1981 survey

^a^adjusted for age and gender

The exclusion of all individuals who reported any pain in either back, shoulder, or neck in 1975 left 5691 individuals. Only 4.8 % of these were later classified in LC3. This diminished the ORs of the regional pain variables from 1981, including headache, in the multivariate analysis. Regional back pain, sleep problems, overweight, and headache, however, still remained significant predictors. The OR for frequent headache diminished from 7.10 to 5.01 and for back pain from 2.99 to 2.32; other changes in OR estimates varied by 20 % at most. In the univariate analyses, all variables except exercise frequency were significant. The amount of missing answers ranged from 4 to 16 %.

### Within-pair analysis

In the main analysis sample (of 8434 individuals) were 2345 twin pairs, of which 802 were monozygotic (MZ), 1401 dizygotic (DZ); 142 pairs had uncertain zygosity, and 3653 individual twins were without their co-twins (due to non-response or exclusion criteria). There were only 161 twin pairs discordant for their classification in LC3 vs. LC1. Of these pairs, 33 were MZ, 118 DZ, and 10 were uncertain-zygosity pairs. Significant associations of predictors with symptom class within pairs were evident for headache, back, neck, and shoulder pain, but not for BMI, sleep quality, or education (Table 3). If the association between the presumed predictor and classification in LC3 depended on familial factors, no association (or at least one much weaker) would exist in this analysis between the predictor variable and LC3. If the association depended specifically on genetic factors, we would presumably find some association among the DZ pairs (who share approximately 50 % of their genes) but not in the MZ pairs (with an almost identical genotype). The associations were generally equally strong in DZ pairs analysed alone, but with some loss of statistical significance due to smaller sample sizes. In MZ pairs, the effects were generally greatly attenuated, and none of the associations within pairs are significant.

**Table 3:** Pairwise analyses of odds ratios (with 95 % confidence intervals) for future classification in latent class 3 (LC3) with FM symptoms (in 1990) in twin pairs discordant for this classification (LC3 vs. LC1). Predictors are assessed from 1981

Predictor	Odds ratios (with 95 % confidence intervals) for LC3 by zygosity					
	All discordant twin pairs		Dizygotic twin pairs		Monozygotic twin pairs	
	OR (95 % CI)	*n*	OR (95 % CI)	*n*	OR (95 % CI)	*n*
back pain (yes/no)	2.7 (1.4–5.2)	117	2.3 (1.1–4.6)	86	5.0 (0.6–42.8)	23
shoulder pain (yes/no)	3.1 (1.3–7.4)	91	8.0 (1.8–34.8)	64	1.0 (0.3–3.5)	23
neck pain (yes/no)	4.0 (1.5–10.7)	92	5.0 (1.4–17.3)	64	2.0 (0.4–10.9)	24
poor sleep	1.4 (0.4–4.4)	144	1.3 (0.3–6.0)	104	1.5 (0.3–9.0)	31
good sleep	1.0 reference category		1.0		1.0	
BMI		143		103		31
overweight	1.9 (1.0–3.8)		1.6 (0.8–3.3)		5.0 (0.6–42.8)	
underweight	0.5 (0.0–5.5)		0.5 (0.0–5.5)		omitted^a^	
normal weight	1.0 reference category		1.0		1.0	
headache (yes/no)	2.1 (1.0–4.1)	132	2.2 (1.0–4.9)	97	2.5 (0.5–12.9)	27

*n* number of twin pairs discordant for each variable, *LC3* those with many fibromyalgia symptoms, *LC1* those with no or few fibromyalgia symptoms

Sums of dizygotic and monozygotic pairs do not equal the sums of all discordant twin pairs, because the zygosity of some pairs remained uncertain

^a^Number of individuals in this category was too small for an adequate analysis

## Discussion

In this simultaneous analysis of several potential predictors for membership in a latent class characterized by having multiple symptoms of fibromyalgia, the strongest predictor was headache, with a dose-dependent effect. Other predictors were back and neck pain, overweight, and sleep problems in this prospective follow-up of a large cohort of adults from the Finnish Twin Cohort, analysed both as individuals and through within-pair analyses.

A strong association between headache and FM has occurred in one cross-sectional study as well, particularly between chronic migraine and chronic tension-type headache (TTH) [22]. A recent large, prospective, population-based study found a bi-directional relationship between chronic daily headache and chronic musculoskeletal complaints (CMSC). In that study, headache at baseline produced a greater risk for widespread CMSC than for non-widespread CMSC [23]. The prevalence of migraine reported in our study is comparable with that of other epidemiological studies [24, 25], and the assessment may therefore be considered reliable. Moreover, a self-report of physician-diagnosed major chronic disease has high validity compared to medical records [26]. Migraine, however, failed to predict FM symptoms. We have no knowledge of the persistence of headache in this group, as the occurrence was assessed only in 1981. Reports of a headache frequency as “daily or nearly daily” or “several times per week” refer to a chronic condition, however. Chronic TTH (referring to our headache category with the highest frequency) may lead to central sensitisation with increased peripheral multi-modal pain sensitivity in skin, muscle, and peripheral nerves [27]. Generalised muscular hyperalgesia is seen in frequent episodic TTH, as well [28].

For TTH, the most commonly reported precipitant is psychological distress [29], while non-specific headache associates with numerous work-related stressors [30]. Long-lasting stressors elevate cytokine levels [31] and cytokines may play a role in the activation of trigger points and in peripheral sensitisation in FM [32–35]. In several studies, treatment of co-occurring trigger points has alleviated generalised pain in FM [36].

Overweight or obesity magnified the risk for FM symptoms markedly depending on the time of the prospective evaluation and the level of the BMI. A mediator of the effect of overweight may be inflammatory activity of the adipose tissue, causing peripheral sensitisation [37, 38]. Probably other mediators exist as well. Another connection between overweight and FM symptoms may be over-activation of the stress system leading to sleep problems and weight gain. Higher stress levels may also be a consequence of stigmatisation in society. Numerous studies have shown an association between psychological distress and subpopulations of FM patients [39–42], but the causal relationship may be bi-directional. This may also be the case with perturbations of the stress system (hypothalamic-pituitary-adrenal axis and autonomic nervous system) [43]. In this study, there was no association between underweight and FM symptoms, while in the HUNT study it was associated with increased risk for chronic WSP [11].

This may be due to the low frequency of underweight subjects in our cohort.

Back pain was a strong predictor, consistent with the results of Forseth and colleagues (1999) and Mikkelsson and colleagues (2008) who studied schoolchildren [2, 3]. As in this study of FM symptoms, local or regional pain states have also proven to be predictors for widespread pain [44]. Repetition of pain stimuli in human pain experiments also leads to increased pain and an enlarged pain area. Chronic pain is related to deficient top-down modulation of pain [45]. Theoretically, long-lasting regional pain could lead to central sensitisation and to widening of the painful area. Indeed, in further analyses, we found that the groups with persistent, either chronic or recurrent, regional pain produced even greater ORs for FM symptoms. These findings are in concordance with the generalised deep-tissue hyperalgesia in patients with chronic low-back and radiating pain after disc herniation [46] and with the generalised mechanical allodynia in patient groups with various types of chronic pain [47]. Moreover, when we excluded the subjects reporting any pain in 1975, the proportion of individuals later classified into LC3 dropped markedly. This suggests slow development over years if not over decades of the FM symptoms and strengthens the finding of the predictive role of regional pain. The increasing predictive power of the pain variables when representing persistent pain agrees well with the central sensitisation theory.

Given the association of sleep disturbance and pain, the effect of sleeping poorly on future risk for FM symptoms was expected and is in concordance with the studies of Mork and Nilsen [10] and Mundal and colleagues [11]. Sleep disturbances also associate with headache [29, 48] and psychological distress in many ways [49, 50].

Conversely to our expectations and to the study of Mork and colleagues (2010) [5], we found no relationship between exercise and FM symptoms. At baseline, subjects in the future high-symptom class were more passive than in the other two classes. In bivariate analyses, both the reported physical activity and reported exercise frequency reduced OR for FM symptoms, but no dose response appeared either in the reported exercise classification or in a more detailed quantitative classification (data not shown). We found no such relationship when analysing the genders separately, either. A reason for this discordance might be that our final sample was smaller than in the study of Mork and colleagues. Second, the dependent variables differed. Third, our primary exercise parameters may be insufficiently accurate. Lastly, other, more powerful variables, regional pain variables and sleep (not included in Mork’s study), diminished the effect of exercise factors in multivariate analyses.

We earlier showed an additive genetic effect behind the distribution of the original twin sample in three FM symptom classes. The small number of discordant pairs and decreasing proportion of MZ pairs among them (compared to the whole sample) are therefore logical. Pain variables associated significantly with future FM-symptoms also within twin pairs. We cannot, however, exclude the predictive power of regional pain problems as being partly dependent on genetic factors. None of the associations in MZ pairs were significant -- perhaps due both to insufficient statistical power and to full adjustment for genetic background. For headache and back pain, MZ point estimates are of the same magnitude as in DZ pairs and in all pairs, consistent with an association with FM-like symptoms independent of family background and genes.

Strengths of this study are the large study population with a relatively good response rate, the prospective setting with a 9- to 15-year follow-up, and the possibility of analysing many relevant predictors simultaneously. Unfortunately, no pain drawing was included in the three questionnaires, so results are not directly comparable with studies on widespread pain. Despite this, and the lack of an FM-question battery before 1990, we were able to make exclusions at baseline on the basis of reported regional pain at multiple sites and frequent analgesic use. The proportion of excluded subjects (13.6 %) reflected the generally estimated WSP prevalence quite well. Moreover, the exclusions, based on other pain assessments in 1975–1980, diminished the proportion of the future FM symptom class by approximately 30 %. Analyses of the subpopulation with no reported pain in either neck, shoulders, or back (and thus with no widespread pain) in 1975 yielded marginally smaller odds ratios for new-onset regional pain.

The two baseline questionnaires from 1975 to 1981 unfortunately included no assessment of anxiety, though it is connected variously with pain [51–53] and would have been valuable to investigate as a possible predictive factor. Nor did we have a formal assessment of depression at baseline. Two recent large prospective population-based studies revealed that anxiety was associated with new-onset WSP [11, 54], while depression had a weaker association in one of them [11] and no association in the other among older adults [54]. However, there is also evidence that depression would follow rather than precede pain [55, 56]. Features such as sensitivity to distress and adaptive coping skills were not assessed in our material either but would have been of interest as well.

The majority of variables were based on self-report, as usual in large epidemiological studies. A trend for overestimation of physical activity [57] is possible and may dilute an association between physical inactivity and FM symptoms. Self-estimated sleep is not as reliable as an objective measurement [58] but is valuable as a reflection of subjective experience [59], and is predictive of morbidity and mortality [60]. Pain ratings are always subjective.

## Conclusions

Headache, back and neck pain, sleeping problems, and high BMI were predictors of FM symptoms, predictors which may be connected. Sleeping problems and high BMI were influenced by familial factors. The intensity and persistence of regional pain was associated with increased risk for FM symptoms.

Heritability plays an important role in FM symptoms, but also in many of the predictors. Therefore that headache and regional pain are predictors independent of family background is an important finding. Further studies must evaluate possibilities of preventing FM by treatment and management of such predictors.

## Additional files

Additional file 1:
**Baseline characteristics of the 8343 subjects in 1975 and 1981 for the whole sample and in relation to latent fibromyalgia symptom classes (assessed in 1990).** (PDF 18 kb) Additional file 2:
**Predictors of fibromyalgia symptoms: univariate analyses.** (PDF 23 kb)

## Abbreviations

BMI: body mass index
CI: confidence interval
FM: fibromyalgia
DZ: dizygotic
MZ: monozygotic
LC: latent class
OR: odds ratio
TTH: tension type headache
WSP: widespread pain
