# Supplementary material for: Predictors of fibromyalgia: a population-based twin cohort study
Source: BMC Musculoskelet Disord. 2016 Jan 15;17:29. doi: 10.1186/s12891-016-0873-6 (PMC4715288; doi:10.1186/s12891-016-0873-6)
Supplement: Additional file 1: — Baseline characteristics of the 8343 subjects in 1975 and 1981 for the whole sample and in relation to latent fibromyalgia symptom classes (assessed in 1990). (PDF 18 kb) [file 12891_2016_873_MOESM1_ESM.pdf]

Additional file 1 Baseline characteristics of the 8 343 subjects in 1975 and 1981 for the whole sample and in relation to latent fibromyalgia symptom classes (assessed in 1990).

	1975				1981			
	All %	LC1 %	LC2 %	LC3 %	All %	LC1 %	LC2 %	LC3 %
age (years)	27.7±7.3	26.5±7.0	28.4±7.6	31.3±7.8				
back pain	21.3	17.0	25.9	36.3	23.2	18.8	28.4	37.3
no back pain	76.4	80.6	72.0	61.0	64.0	71.1	56.1	40.7
missing data	2.3	2.4	2.0	2.7	12.7	10.1	15.6	22.0
shoulder pain	6.0	4.2	8.0	11.4	12.6	9.0	17.1	23.0
no shoulder pain	91.6	93.4	89.8	85.9	66.9	73.4	59.1	47.3
missing data	2.4	2.4	2.1	2.7	20.5	17.6	23.8	27.9
neck pain	7.7	5.0	11.1	14.9	13.6	10.1	17.6	25.4
no neck pain	89.9	92.5	86.8	82.4	66.9	73.2	59.6	46.7
missing data	2.4	2.5	2.2	2.7	20.5	16.7	22.8	27.9
poor sleep	3.5	2.9	4.0	6.7	4.5	3.1	5.7	9.9
good sleep	93.8	94.3	93.6	90.1	91.0	92.5	89.7	84.7
missing data	2.7	2.8	2.4	3.1	4.5	4.4	4.6	5.4
BMI								
≥ 30	1.6	1.3	1.9	3.1	2.6	2.1	3.1	4.6
25,0 – 29,9	14.4	12.0	16.2	25.6	19.8	16.9	22.4	31.1
18,5 – 24,9	74.9	76.8	74.1	63.9	69.5	72.2	67.5	57.1
< 18,5	6.1	6.9	5.1	4.0	3.6	4.4	2.6	1.1
missing data	3.0	3.0	2.8	3.4	4.6	4.4	4.5	6.0
education (years)	8.4±2.9	8.6±3.0	8.1±2.8	7.3±2.2	8.7±3.3	9.0±3.4	8.4±3.1	7.4±2.4
missing data	2.3	2.4	1.9	2.6	4.0	3.8	3.9	5.4
physical activity								
passive	53.1	51.9	53.7	59.3	46.9	45.6	48.2	51.6
average	31.6	31.4	32.9	28.4	34.9	34.9	35.5	32.6
active	13.0	14.3	11.2	9.7	14.2	15.6	12.4	10.4
missing data	2.3	2.4	2.2	2.6	4.0	3.9	3.8	5.4
exercise frequency/month								
at most 2 times	29.3	28.4	30.6	31.1	22.8	22.3	23.1	25.9
3-10 times	47.9	47.4	48.9	47.6	51.5	51.4	52.5	48.0
at least 11 times	17.4	18.7	15.6	14.1	19.8	20.6	18.5	18.1
missing data	5.4	5.5	4.9	7.1	5.9	5.7	5.9	8.0
smoking								
current	30.5	29.9	31.1	32.9	25.5	24.4	26.8	29.0
former	15.1	14.7	15.9	14.9	20.1	19.9	20.8	18.6
occasional	3.5	3.5	3.9	2.4	3.0	3.1	2.8	2.6
never	48.5	49.4	47.0	47.0	46.6	47.9	44.8	43.3
missing data	2.4	2.4	2.1	2.9	4.9	4.7	4.8	6.6
migraine					8.7	7.6	9.4	13.6
no migraine	n.a.				87.1	88.3	86.3	80.4
missing data					4.3	4.1	4.2	6.0
headache frequency								
daily – some days/week					2.8	1.6	3.9	7.9
once/week – once/mo					27.1	24.2	31.5	33.1
some times / year					42.7	42.8	43.4	39.3
never	n.a.				23.1	27.4	17.0	13.9
missing data					4.2	4.1	4.2	5.9

LC1 = latent class 1 with few or no symptoms, LC2 = latent class 2 with some symptoms, LC3 = latent class 3 with a high frequency of FM symptoms in 1990
