# Supplementary material for: Predictors of fibromyalgia: a population-based twin cohort study
Source: BMC Musculoskelet Disord. 2016 Jan 15;17:29. doi: 10.1186/s12891-016-0873-6 (PMC4715288; doi:10.1186/s12891-016-0873-6)
Supplement: Additional file 2: — Predictors of fibromyalgia symptoms: univariate analyses. (PDF 23 kb) [file 12891_2016_873_MOESM2_ESM.pdf]

Additional file 2. Predictors of fibromyalgia symptoms: univariate analyses.

	Number of subjects by variable 1975	OR (95% CI) LC2 1975 variable data	OR (95% CI) LC3 1975 variable data	Number of subjects by variable 1981	OR (95% CI) LC2 1981 variable data	OR (95% CI) LC3 1981 variable data
age [a)] (years)	8 343	1.03 (1.02 - 1.04)	1.08 (1.07 – 1.09)	8 343	1.03 (1.02 - 1.04)	1.08 (1.07 – 1.09)
gender [b)] (female/male)	8 343	1.22 (1.11 - 1.35)	1.20 (1.02 – 1.41)	8 343	1.22 (1.11 - 1.35)	1.20 (1.02 – 1.41)
back pain (yes/no)	8 149	1.66 (1.48 - 1.87)	2.55 (2.14 – 3.04)	7 281	1.94 (1.72 - 2.17)	3.40 (2.82 – 4.10)
shoulder pain (yes/no)	8 146	1.85 (1.51 - 2.26)	2.49 (1.89 – 3.29)	6 636	2.24 (1.94 – 2.60)	3.51 (2.82 – 4.36)
neck pain (yes/no)	8 144	2.15 (1.80 - 2.57)	2.65 (2.06 – 3.40)	6 718	2.03 (1.76 – 2.34)	3.44 (2.79 – 4.24)
poor sleep		1.37 (1.06 - 1.78)	2.24 (1.58 – 3.16)		1.85 (1.47 – 2.33)	3.20 (2.37 – 4.33)
good sleep	8 116	1.00 (reference category)	1.00	7 966	1.00	1.00
BMI (kg/m-squared)						
≥ 30		1.35 (0.92 - 1.98)	2.05 (1.24 – 3.39)		1.47 (1.08 – 1.98)	2.12 (1.40 – 3.22)
25 – 29.9		1.30 (1.13 - 1.51)	1.90 (1.55 – 2.34)		1.39 (1.23 – 1.58)	1.96 (1.62 – 2.38)
18.5-24.9		1.00 (reference category)	1.00		1.00	1.00
< 18.5	8 056	0.78 (0.63 - 0.97)	0.88 (0.59 – 1.33)	7 913	0.63 (0.47 – 0.84)	0.38 (0.19 – 0.78)
education (years)	8 134	0.94 (0.93 - 0.96)	0.84 (0.81 – 0.87)	7 754	0.95 (0.93 – 0.96)	0.85 (0.82 – 0.88)
physical activity						
passive		0.99 (0.89 - 1.11)	1.31 (1.09 – 1.57)		1.04 (0.93 – 1.15)	1.22 (1.02 – 1.46)
moderate		1.00 (reference category)	1.00		1.00	1.00
active	8 148	0.78 (0.67 - 0.92)	0.80 (0.60 – 1.08)	8 007	0.80 (0.68 – 0.93)	0.72 (0.55 – 0.95)
exercise frequency./month						
1-2 times		1.07 (0.96 - 1.20)	1.19 (0.99 – 1.43)		1.03 (0.91 – 1.16)	1.29 (1.06 – 1.57)
3-10 times		1.00 (reference category)	1.00		1.00	1.00
> 11 times	7 889	0.81 (0.71 - 0.93)	0.76 (0.60 – 0.97)	7 849	0.87 (0.77 – 0.99)	0.92 (0.74 – 1.14)
smoking						
current		1.21 (1.08 - 1.36)	1.44 (1.19 – 1.74)		1.32 (1.17 – 1.49)	1.70 (1.39 – 2.08)
former		1.16 (1.01-1.34)	1.06 (0.83 – 1.35)		1.19 (1.04 – 1.35)	1.15 (0.92 – 1.45)
occasional		1.23 (0.95-1.59)	0.80 (0.47 – 1.33)		1.08 (0.81 – 1.45)	1.11 (0.67 – 1.85)
never	8 146	1.00 (reference category)	1.00	7 936	1.00	1.00
migraine (yes/no)		n.a.	n.a.	7 986	1.17 (0.99 – 1.39)	1.72 (1.35 – 2.20)
headache frequency						
many /week					4.33 (3.15 – 5.95)	12.16 (8.02 – 18.42)
1 - 4 /month					2.19 (1.89 – 2.53)	3.18 (2.45 – 4.13)
some/year					1.69 (1.48 – 1.93)	2.02 (1.58 – 2.59)
never		n.a.	n.a.	7 767	1.00 (reference category)	1.00

The reference category for the outcome is LC1: those with no or very few fibromyalgia symptoms [12]. Odds ratios and 95% confidence intervals are provided for LC2 (some fibromyalgia symptoms) and LC3 (those with many fibromyalgia symptoms), based on latent class (LC) analyses. Risk factors are assessed from 1975 and 1981 with the exception of migraine and headache frequency from 1981 only.
[a)] adjusted for gender [b)] adjusted for age
All the other variables were adjusted for both age and gender.
